# Preparation and Characterization of an Antibrowning Nanosized Ag-CeO_2_ Composite with Synergistic Antibacterial Ability

**DOI:** 10.3390/ma16165505

**Published:** 2023-08-08

**Authors:** Min Yang, Mi Liu, Genli Shen, Yan Gong, Zhen Wang, Daiyu Ji, Jianqiang Li, Min Yuan, Qi Wang

**Affiliations:** 1 School of Chemistry and Biological Engineering, University of Science and Technology Beijing, Beijing 100083, China; yangmin@ustb.edu.cn (M.Y.); lijq@sas.ustb.edu.cn (J.L.); 2 CAS Key Laboratory of Standardization and Measurement for Nanotechnology, National Center for Nanoscience and Technology, Beijing 100190, China; liumi@nanoctr.cn (M.L.); shengl@nanoctr.cn (G.S.); gongyan2019@nanoctr.cn (Y.G.); wangzh@nanoctr.cn (Z.W.); 3 Inner Mongolia XinYu Rare Earth Functional Materials Co., Ltd., Baotou 014060, China; jidaiyu@163.com; 4 Bgrimm Mtc Technology Co., Ltd., Beijing 102628, China

**Keywords:** antibrowning, Ag-CeO_2_ composite, silver ion release rate, hydroxyl radicals, antibacterial property

## Abstract

Nanosized Ag and CeO_2_ particles obtained through the hydrothermal method were physically mixed to obtain composite antibacterial agents. The comparative experiments of antibacterial properties showed that the antibacterial activity of the nanocomposites was improved compared to the nanoparticles alone, which indicated that the synergistic antibacterial effect existed between Ag and CeO_2_. On the one hand, ICP-MS results showed that the existence of CeO_2_ suppressed the silver ion release rate and provided the composite with the ability of antibrowning; on the other, EPR data indicated that more hydroxyl radicals (·OH) were generated by the interfacial interaction between nanosized Ag and nanosized CeO_2_. Hence, for the Ag-CeO_2_ composite antibacterial agent, hydroxyl radicals played an important role in causing bacterial death.

## 1. Introduction

The abuse of traditional antibiotics has exacerbated the selective evolution of bacteria and poses a great threat to human health [1,2]. Nowadays, the advent of nanomaterials and their introduction into the biological field for the treatment of multiple diseases has provided new ideas for the development of superior antibacterial agents [3,4]. Among these nanomaterials, silver nanoparticles stand out because of their high temperature resistance and their broad-spectrum antibacterial and drug resistance [5,6,7,8]. However, silver nanoparticles suffer from poor dispersion, the quick release of Ag^+^, poor preservation and instability [9]. The addition of the rare earth element cerium oxide was a feasible way to overcome these flaws. There were some reports which indicated that CeO_2_ could help to improve antibacterial property, reduce cytotoxicity and enhance the dispersion stability of Ag nanoparticles [10,11,12,13,14]. The synthetic methods of combining silver with ceria composites have been widely explored, including impregnation, coprecipitation, green synthesis, laser ablation, wet chemical, microwave assistance and so on [12,15,16,17,18,19]. The relationship between the structure and performance of silver–cerium oxide composites has also been studied. Neal et al. [18] found that the distinct silver-modified formulations of redox-active nanoscale cerium oxide influenced antiviral activities. A greater density of silver phases on ceria surfaces led to a greater total interface area, providing potent antiviral activity against coronavirus. In order to improve the interface area and metal dispersion, much work had been completed recently [20,21]. The synergistic antibacterial activity of silver with cerium oxide composites was also discussed [20,21,22,23,24]. Li et al. [20] reported that a Ag/CeO_2_ nanocomposite prepared via selective laser welding in liquid had obvious composite interfaces. The results show that the Ag/CeO_2_ nanocomposite had a better antibacterial activity than pure Ag and pure CeO_2_, and the sterilization rate was boosted 2.93 and 2.99 times, respectively. Muhammad et al. [21] found that the ultrafine noble metal (Ag, Au, and Pt) nanocrystals can be homogeneously immobilized onto the surface of CeO_2_ nanospheres by employing the innate reductive potential of CeO_2_. The composites showed superior anti-inflammatory activities compared to single metal NPs. However, it is difficult to simply and cleanly clarify the synergistic antibacterial mechanism of silver–cerium oxide composites due to the introduction of many complex influencing factors during experiments—for example, the change in nanoparticles size, dispersion stability, new reactants after composite reactions, and so on.

In this work, the silver–cerium oxide composite was prepared via the simple physic mixture method, which makes it easy to study the synergistic antibacterial mechanism. At the same time, the dispersion stability and antibrowning ability of CeO_2_ coupled with nano-silver were also inspected. The antimicrobial activity of silver-cerium oxide composite material and individual nanoparticles against *Escherichia coli* ATCC 25922 (*E. coli*) was evaluated. The synergistic antibacterial mechanism of CeO_2_ coupled with nano-silver was further discussed. It was expected that reactive hydroxyl radicals could play a key role in the inactivation of *E. coli.*

## 2. Experiment

### 2.1. Synthesis of the Nanosized Ag, CeO_2_ and Ag-CeO_2_ Composite

The typical synthesis process of silver nanoparticles (Ag NPs) was as follows: 150 mL of AgNO_3_ (0.5 mmol/L) aqueous solution was prepared, and 50 mL of sodium citrate (1 mmol/L) and 50 mL of polyvinylpyrrolidone (PVP-K30, 1 g/L) were added after stirring for 10 min. Then, 0.2 mL of NaBH_4_ (5 mmol/L) was added with continuous stirring. After 15 min, the reaction mixture was reacted at 160 °C under hydrothermal conditions for 3 h to obtain a Ag NP sample. The product was washed several times with distilled water and centrifuged at 9500 rpm for 30 min each time to get the pure Ag NPs.

The nanosized CeO_2_ was also synthesized using the hydrothermal method: 1 g of PVP was first dissolved in 50 mL of deionized water, 20 mL of Ce(NO_3_)_3_·6H_2_O (0.12 mol/L) was added into the above solution with stirring for 30 min, and then 0.5 mL of ammonia (25%wt) was added and stirred for half an hour. The above solution was transferred to a 100 mL hydrothermal reactor and was reacted at 165 °C for 24 h to obtain well-dispersed CeO_2_ NPs.

The Ag NPs and CeO_2_ NPs obtained above were individually washed three times with deionized water and then were mixed according to several certain concentrations. The composite antibacterial agent was obtained after 1 h of mechanical stirring at room temperature with a speed of 500 rpm and being left to sit for 12 h, labeled as Ag-CeO_2_. The selection of the concentration values of the two nanoparticles in the composite antibacterial agent was based on the antibacterial performance when they existed alone, and the minimum antibacterial concentration was selected as the initial concentration value.

### 2.2. Characterization

The crystal structure of all of the samples was analyzed via X-ray diffraction at room temperature (XRD, PANalytical Empyrean), in the 2 θ range of 2°–90°. The surface morphologies and size of the samples were examined under a high-resolution transmission electron microscope (TEM, Jeol, JSM-6610 operating at 200 V). The silver ion release rate was obtained using an inductively coupled plasma mass spectrometer (ICP-MS). The optical properties of nanomaterials were monitored using a Cary 50 UV-Vis spectrophotometer. Electron paramagnetic resonance (EPR) spectra were obtained using a Bruker EMX PLUS spectrometer. The settings for the EPR spectrometer were center field 3500.00 G, resonance frequency 9.823 GHz, and power 6.325 mW. The *E. coli* ATCC 25922 (Gram-negative) strain was chosen for this experiment.

### 2.3. Antibacterial Test

The *E. coli* strain was chosen for this experiment. All materials were autoclaved at 121 °C for 20 min before using. Bacterial suspension without the bacteriostatic agent was used as the control group. In this process, 0.1 mL of *E. coli* suspension was injected into 0.9 mL of sterile water, and the prepared Ag, CeO_2_ and Ag-CeO_2_ nanoparticles were added into the system, respectively. After adding nanoparticles for 120 min, a 0.5 mL bacterial suspension was extracted and plated on LB agar plate. Viable cell counts were determined after 24 h of incubation at 37 °C. All experiments were repeated in triplicate.

The dissolution kinetics were examined using ultrafiltration tubes for Ag NPs and Ag-CeO_2_ NPs. At intervals of 10, 30, 60 and 120 min after the sample solution was centrifuged and re-dispersed in water, 4 mL of pure Ag NPs and the Ag-CeO_2_ NP solution with a uniform original silver concentration of 35 µg/mL were transferred into ultrafiltration tubes, respectively. After ultrafiltration centrifugation, the lower liquid of the ultrafiltration tube was taken for determination. The dissolved amount of silver ions was determined via ICP-MS.

## 3. Result and Discussion

**Figure 1:**
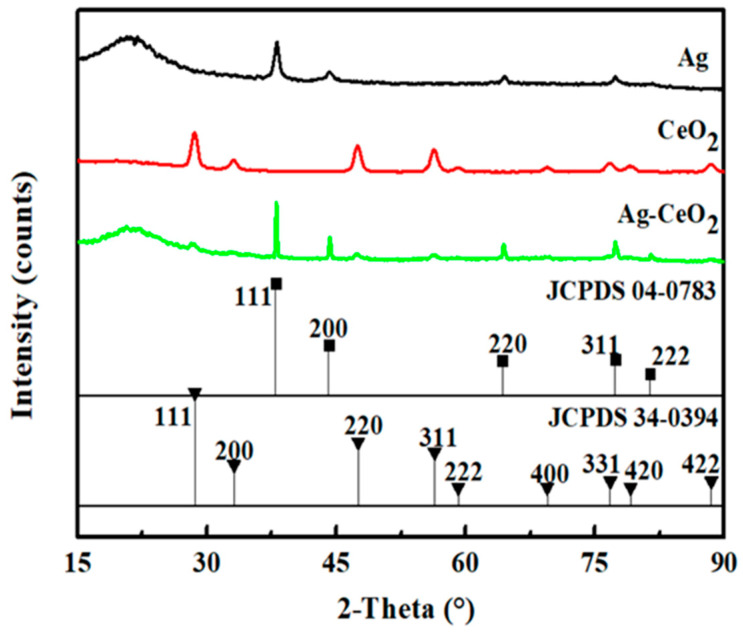
XRD patterns of nanosized Ag, CeO_2_ and the Ag-CeO_2_ composite.

The crystal structure of the as-synthesized Ag, CeO_2_ and Ag-CeO_2_ composite was characterized using XRD. Figure 1 shows that there are no impurity peaks in each sample, which indicates that the crystal form is intact. The spectra of the Ag NPs match the diffraction data card number JCPDS 04-0783 corresponding to the face-centered cubic (FCC) structure, while the spectra of the CeO_2_ NPs match the JCPDS 34-0394 for the cubic fluorite structure. There is no obvious change in the peak position of the Ag-CeO_2_ composite compared with individual nanoparticles, due to the simple physical mixing for Ag and CeO_2_ NPs.

**Figure 2:**
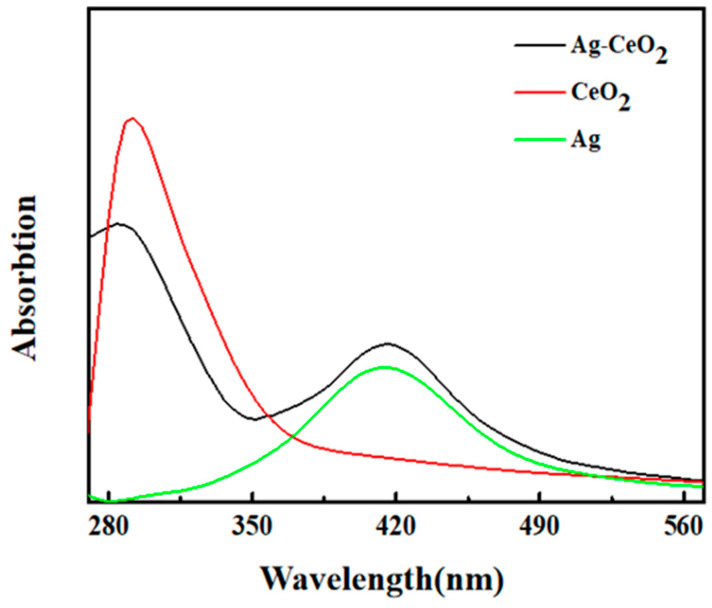
Ultraviolet-visible absorption spectra of nanosized Ag, CeO_2_ and Ag-CeO_2_.

The peak band at about 410 nm corresponds to the characteristic surface plasmon resonance (SPR) of Ag NPs (Figure 2). The UV absorption peak of CeO_2_ is about 292 nm. In the absorption band of the Ag-CeO_2_ composite, the mixing of Ag and CeO_2_ led to an 8 nm blue shift compared with that of pure nanosized CeO_2_ and a 6 nm red shift compared with that of pure nanosized Ag. These shifts in peak position can be explained by the electrostatic potential effect and the quantum effect of nanoparticles caused by the valence state transfer of cerium ions [15,16]. The interface interaction between Ag NPs and CeO_2_ NPs has an effect on the antibacterial properties of the Ag-CeO_2_ composite.

**Figure 3:**
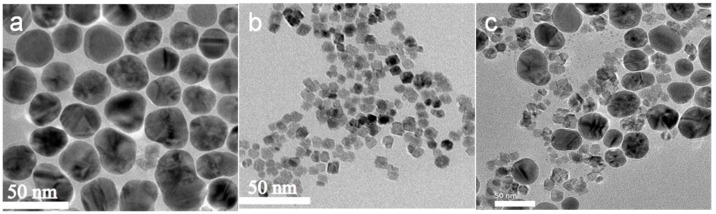
TEM images of nano-sized Ag (**a**), CeO_2_ (**b**) and the Ag-CeO_2_ composite (**c**).

The morphology of the Ag NPs, CeO_2_ NPs and Ag-CeO_2_ composite was studied via transmission electron microscopy. The results are shown in Figure 3. Figure 3a provides the transmission electron microscope image of Ag NPs with particle size about 40 nm. The particle shape is spherical, and the size distribution is uniform. Figure 3b provides the TEM image of CeO_2_ with an average particle size of 15 nm. It can be seen that the dispersion of the sample particles is good and the shape is regular. Figure 3c presents the Ag-CeO_2_ composite, which is prepared via mechanical stirring at room temperature, and it can be seen that the overall particle morphology hardly changes with the continuous contact of the two different nanoparticles. The nanosized Ag and nanosized CeO_2_ are arranged in interval distribution. The concentration of CeO_2_ in the mixture is much higher than that of Ag. Large-sized Ag nanoparticles are surrounded by small-sized CeO_2_ nanoparticles.

**Figure 4:**
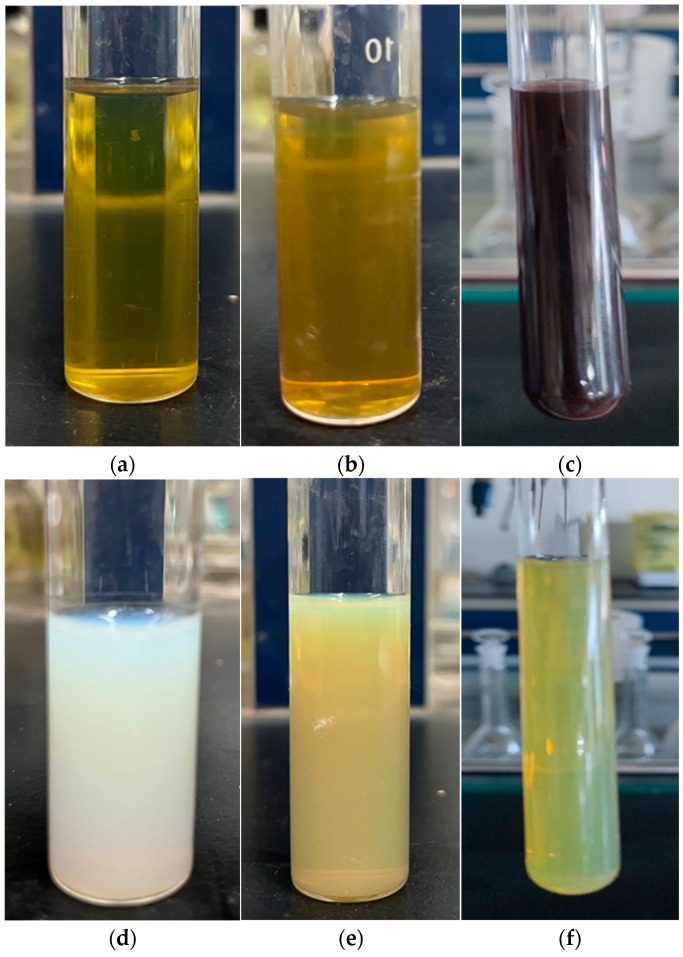
Photographs of nano-sized Ag (**a**), the Ag left to sit for one week (**b**), the Ag left to sit for three months (**c**), nano-sized CeO_2_ (**d**), the Ag-CeO_2_ composite (**e**), and the Ag-CeO_2_ composite left to sit for three months (**f**), respectively.

Figure 4 provides pictures of the prepared nano-antibacterial particles. Figure 4a depicts the newly prepared Ag NPs with a concentration of 35 µg/mL; Figure 4b depicts the Ag NPs after one week. Figure 4c presents the Ag NPs after three months. It can be seen that the color of Ag NPs changed slightly from a transparent bright yellow to a translucent yellow after one week of sealed storage in indoor natural light. After three months, it became brown. It can be seen from Figure 4d that the CeO_2_ NPs with the concentration of 2800 µg/mL are translucent and light white. Figure 4e is a photo of the mixture of the Ag NPs and CeO_2_ NPs, and the composite particles are translucent yellow. Moreover, when the Ag-CeO_2_ composite particles were left to sit for three months, there was no color change and no stratification from Figure 4f.

It can be seen from the monitoring results of the color change of the samples over a long time period that the individual nanosized Ag particle slurry will brown. However, no browning occurred when nanosized Ag particles were mixed with nanosized CeO_2_ particles. When nanosized Ag particles existed alone in water, they underwent a slow oxidation reaction with the oxygen in water and the container bottle. The generated silver oxide was brown, which will affect its application in some areas where color change is not allowed. The CeO_2_ particles have an antioxidant function. When nanosized Ag particles and nanosized CeO_2_ particles are uniformly dispersed and closely contacted with each other, the external oxygen molecules will be adsorbed on the surface of CeO_2_ particles. Even if Ag is oxidized by oxygen atoms into silver ions, silver ions will be immediately reduced by trivalent cerium on the surface of the cerium oxide, thus preventing it from becoming silver oxide [21].

**Figure 5:**
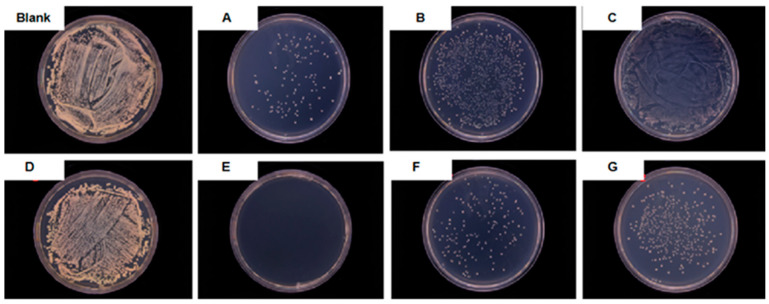
Antibacterial activity of a blank plate and nano-sized Ag, CeO_2_ and the Ag-CeO_2_ composite with different concentrations.

The bactericidal properties of the Ag NPs, CeO_2_ NPs and Ag-CeO_2_ composite were measured, and the results are displayed in Figure 5 and Table 1.

In order to study the antibacterial properties of Ag NPs, different concentrations were screened. Table 1 lists three representative concentrations, which are 35 µg/mL, 17.5 µg/mL and 8.75 µg/mL, respectively. When the concentration of Ag NPs was 8.75 µg/mL, the antibacterial performance was almost zero (Figure 5C, Table 1 sample C), the bacteria multiplied on a large area of the surface and were connected together, making it impossible to count the colonies, and so the antibacterial rate was defined as being close to zero; for 17.5 µg/mL, the antibacterial rate was 70% (Figure 5B, Table 1 sample B); for 35 µg/mL, it was up to 96% (Figure 5A, Table 1 sample A). Obviously, for the individual Ag NPs, with a continuous reduction in the concentration, the antibacterial performance gradually deteriorated, and finally there was no antibacterial effect. This proved that the antibacterial effect was concentration-dependent. Therefore, establishing how to improve its antibacterial ability in the case of a very low silver concentration is very meaningful.

In this study, the antibacterial properties of nanosized CeO_2_ particles with different concentrations were also studied. When the particle concentration decreased to 2800 µg/mL, the antibacterial rate was close to zero (Figure 5D, Table 1 sample D). Therefore, 2800 µg/mL was chosen as the final concentration of CeO_2_ NPs in this study.

After mixing the Ag NPs with a concentration of 70 µg/mL, 35 µg/mL and 17.5 µg/mL, respectively, into the CeO_2_ NPs with a concentration of 5600 µg/mL in an equal volume, the concentration of the two substances was halved, and three different Ag-CeO_2_ composites were obtained, corresponding to E, F, and G in Figure 5 and Table 1. The concentration of Ag NPs in the composite particles aqueous dispersion was 35 µg/mL, 17.5 µg/mL and 8.75 µg/mL, respectively, meanwhile the concentration of the CeO_2_ NPs was 2800 µg/mL.

It can be seen that when both sample C and sample D without antibacterial ability were mixed, their mixture (Figure 5G, Table 1 sample G) produced antibacterial properties, and the antibacterial rate reached 85%. When the composite contained Ag NPs at a concentration of 17.5 µg/mL and CeO_2_ NPs at a concentration of 2800 µg/mL, its antibacterial rate became 95%—compared with Ag NPs at a concentration of 17.5 µg/mL (Figure 5B, Table 1 sample B) alone, the antibacterial rate was increased by 35.7%. The antibacterial property of the Ag-CeO_2_ composite was significantly improved through the mixing of the two. The antibacterial rate of the Ag NPs (35 µg/mL) was 96% (Figure 5A, Table 1 sample A). Under the same Ag NP concentration, when the concentration of CeO_2_ NPs in the slurry was 2800 µg/mL, the antibacterial rate was close to 100% (Figure 5E, Table 1 sample E). The antibacterial rate of sample E, which will does not turn brown after being left to sit for three months, is better than that of sample A, which does turn brown after three months. The experimental results show that there is a synergistic antibacterial effect between Ag NPs and CeO_2_ NPs.

**Figure 6:**
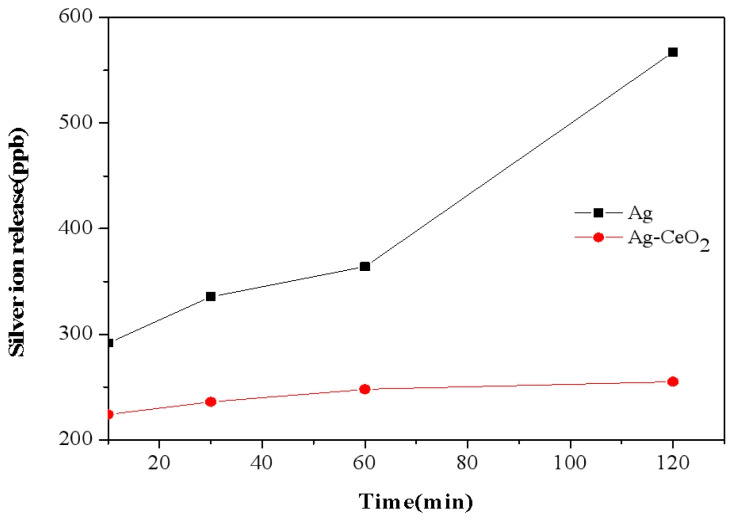
Silver ion release rate of the nano-sized Ag and Ag-CeO_2_ composite.

Figure 6 shows the silver ion release rate for Ag NPs (Figure 5A, Table 1 sample A) and Ag-CeO_2_ NPs (Figure 5E, Table 1 sample E). It is worth noting that the silver ion release rate of pure Ag NPs was 0.23 ng s^−1^, while that of Ag-CeO_2_ was only 0.16 ng s^−1^. Compared with Ag NPs, the silver ions for the Ag-CeO_2_ composite show a certain slow-release effect. This phenomenon suggested that the presence of cerium oxide slowed the silver ion release rate, which contributes to the antibacterial permanence of the material.

**Figure 7:**
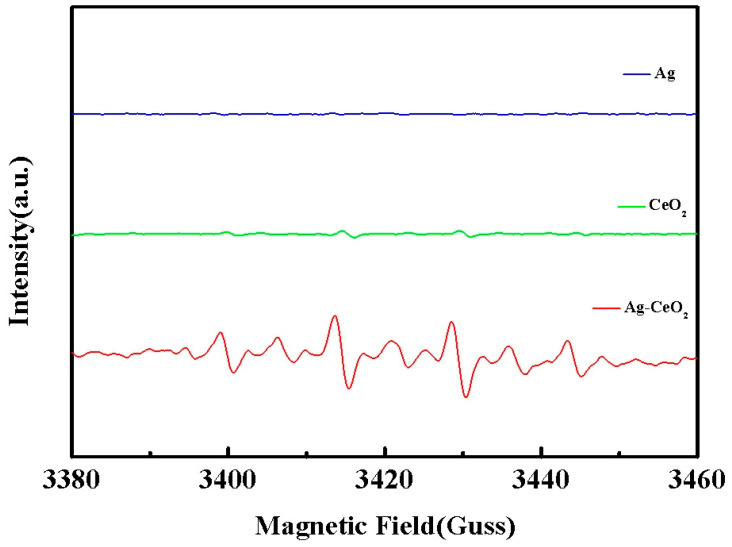
EPR spectra of nano-sized Ag, CeO_2_ and Ag-CeO_2_ recorded at ambient temperature in aqueous dispersion.

Pal et al. [25] have indicated that the strong oxidation of hydroxyl radicals (·OH) in reactive oxygen species is an effective ingredient in the bactericidal process. Thus, the analysis of hydroxyl radicals in Ag, CeO_2_ and Ag-CeO_2_ composites through the electron paramagnetic technique is shown in Figure 7. Since ·OH is very unstable in the solution environment, 5,5-dimethyl-1-Pyrrole-N epoxides (DMPO) are usually used as trapping agents for the determination of hydroxyl radicals. Figure 7 illustrates the EPR spectra of the DMPO-·OH spin adduct. The four characteristic peaks are clearly observed in both CeO_2_ and Ag-CeO_2_ patterns. In addition, the intensities of the ·OH characteristic quartet peaks in the Ag-CeO_2_ composite are enhanced compared with those of CeO_2._ This result supports the following speculation: the interaction of the Ag-CeO_2_ complex produces more oxygen vacancies, leading to the change in the hydroxyl radical level, which is beneficial for the antibacterial effect [26].

**Figure 8:**
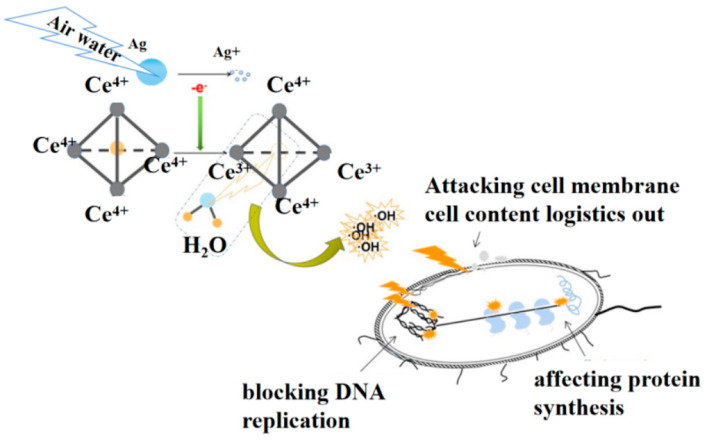
Synergistic antibacterial mechanism of nano-sized Ag-CeO_2_ composite.

Based on the above test results, on one hand, the existence of CeO_2_ suppressed the silver ion release rate, and on the other, the stimulation of silver nanoparticles increases reactive oxygen species production from the composite. Therefore, the possible synergistic antibacterial process in the Ag-CeO_2_ composite is elaborated in Figure 8. Firstly, the nanosized Ag was oxidized into silver ions in the air and water environment. During this process, the lost electrons were transferred to the surface of CeO_2_ [21,22,27]. The electronic conversion between Ce^4+^ and Ce^3+^ stimulated the generation of abundant oxygen vacancies, which oxidized water molecules to promote the increase in hydroxyl radical content. They demonstrated high reactivity and accumulated excessive reactive oxygen species. The abnormal accumulation of reactive oxygen species in cells caused oxidative stress, leading to intracellular changes in protein synthesis and blocking DNA replication, cell senescence and apoptosis [28].

## 4. Conclusions

In this work, the 40 nm nanosized Ag and 15 nm nanosized CeO_2_ were synthesized separately. Their composite particles were prepared via a special mixing process. The antibacterial properties of nanosized Ag, nanosized CeO_2_ and composite particles were compared and tested. It was found that the antibacterial ability of the NPs was significantly enhanced after mixing. When the small CeO_2_ particles were surrounded and dispersed around the large Ag particles, it prevented the browning of silver, meaning that the composite antibacterial agent had the characteristics of no discoloration.

In addition, we found that metal ions were released in a sustained manner, and the production of free radicals increased in the composite antibacterial agent. This indicated that the effect of reactive hydroxyl radicals on the antibacterial process was dominant. This provided a theoretical basis for selecting suitable and efficient antibacterial agents in future research.

## Figures and Tables

**Table 1:** Antibacterial results of Ag, CeO_2_ and the Ag-CeO_2_ composite.

Samples	Blank	A	B	C	D	E	F	G
**Ag**	0	35	17.5	8.75	0	35	17.5	8.75
**CeO_2_** **Concentration (µg/mL)**	0	0	0	0	2800	2800	2800	2800
**Antibacterial Rate (%)**	≈0	96	70	≈0	≈0	≈100	95	85

## Data Availability

The datasets used or analyzed during the current study are availablefrom the corresponding author upon reasonable request.
